# The feasibility of a search filter for the adverse effects of nondrug interventions in MEDLINE and Embase

**DOI:** 10.1002/jrsm.1267

**Published:** 2017-10-11

**Authors:** Su Golder, Kath Wright, Yoon K. Loke

**Affiliations:** ^1^ Department of Health Sciences University of York York YO10 5DD UK; ^2^ CRD University of York York YO10 5DD UK; ^3^ Norwich Medical School University of East Anglia Norwich NR4 7TJ UK

**Keywords:** Adverse Effects, Complications, Information storage and retrieval, Literature searching, Medline

## Abstract

Authors and indexers are increasingly including terms for adverse drug effects in the titles, abstracts, or indexing of records in MEDLINE and Embase. However, it is not clear if this is the same for studies with nondrug adverse effects data.

We therefore assessed the feasibility of using adverse effects terms when searching MEDLINE or Embase to retrieve papers of nondrug adverse effects.

A collection of papers that reported data on nondrug adverse effects was sought from included studies of systematic reviews. Each included study was analysed to ascertain whether the corresponding record in MEDLINE and Embase included adverse effects terms in the title, abstract, or indexing.

From 9129 records screened from DARE, 30 reviews evaluating nondrug adverse effects met our inclusion criteria. From these, 635 unique papers were included in our analysis.

Sensitive searches for adverse effects required generic and specific named adverse effects terms using the title, abstract, and indexing. Records relating to surgical interventions were more likely to contain adverse effects terms than records relating to nonsurgical interventions. Using any adverse effects terms in the title, abstract or indexing in MEDLINE and Embase would have identified an average of 94% of papers on surgical adverse effect interventions per systematic review and 72% of papers on nonsurgical adverse effects per systematic review.

Hence, while a generic nondrug adverse effect search filter may not yet be feasible, a filter for the adverse effects of surgical interventions may be within reach.

## 1 INTRODUCTION

An important part of any systematic review is the process of searching for studies, which needs to be thorough, transparent, and reproducible.^1^ This is usually conducted on a number of databases (such as MEDLINE and Embase) as well as nondatabase resources (such as reference checking). Systematic reviews should use search strategies that aim to identify as many relevant papers as possible. Searches need to be highly sensitive and aimed at identifying all the possible studies on a particular topic. However, highly sensitive searches are often associated with low precision, whereby, lots of irrelevant records are also identified. To improve efficiency, search filters with predefined combinations of search terms have been developed to identify topics (such as paediatrics^2^, ^3^ or research designs such as randomised controlled trials^4^) that are of wide interest. However, searching for information on adverse effects is challenging because adverse effects are often secondary or even tertiary outcomes and the terminology used can be inconsistent.

As studies containing adverse effects data are particularly difficult to search for, creating a search filter in this area would be highly desirable. Although any intervention (be it drug, device, surgical, or behavioural) can have adverse effects, adverse effects search filters have focused primarily on drug interventions^5^, ^6^, ^7^, ^8^, ^10^. There is increasing recognition that these filters do not perform well for nondrug adverse effects^11^, ^12^—such as medical devices and surgical interventions. It has therefore been proposed that different combinations of search terms would be required.^12^, ^13^ However, the feasibility of creating search filters for the adverse effects of nondrug interventions has yet to be established. If adverse effects search terms are present in the bibliographic records, these terms could potentially be used in a database search strategy or used to create a search filter.^12^

We aimed to assess the feasibility of creating validated search filters for nondrug interventions in MEDLINE and Embase. This is the first step towards creating nondrug adverse effects search filters or enabling searchers to create their own combinations of adverse effects search terms for their review. Although feasibility can be subjective, for our purposes we adopted a definition of a combination of terms that would identify greater than 90% of relevant records indexed.^14^

## 2 METHODS

A collection of papers that reported data on the frequency of adverse effects of nondrug interventions was sought from the included studies of systematic review of adverse effects.

### 2.1 Selection of systematic reviews

Systematic reviews of adverse effects were identified by manually screening all records published in 2014 in the Database of Abstracts of Reviews of Effects (DARE) (via the Centre for Reviews and Dissemination web site, April 2015). No automated search strategy was implemented, as previous research has indicated that even very broad search strings would miss relevant records.^15^ The DARE database was chosen because it was the most accessible major collection of systematic reviews of health care interventions. DARE was compiled through rigorous monthly searches of bibliographic databases, including MEDLINE and Embase, as well as handsearching of key journals, grey literature, and regular searches of the Internet. It also contains all Cochrane reviews, both new and updated. DARE ceased production in March 2015 but continues to be available in archive format. A systematic review was considered eligible for inclusion if

- Adverse effect(s) for a nondrug intervention were the primary outcome.
- Generic adverse effects search terms or specified named adverse effects search terms had not been used by the review authors. This enabled an unselected cohort to be built, where relevant articles were not chosen because of the presence of adverse effects terms. Typically, such reviews would have relied on search terms for population/condition and intervention only.
- The search included either handsearching or reference checking in addition to database searches. This may identify additional records available in MEDLINE and Embase but not identified by the search strategy used by the reviewers.

Criteria b and c were applied in previous research on adverse drug effects searching by Derry et al^16^ and Golder and Loke.^5^ Maintaining this consistency with previous research methods also ensured that we were able to make direct comparisons with such research. The author and another researcher independently screened titles and abstracts in DARE and selected full articles for inclusion. Any discrepancies between the researchers were resolved by discussion and consensus.

### 2.2 Selection of primary studies

The included references from the systematic reviews were then used to create a gold standard set of records. Full‐text articles were checked to confirm the presence of adverse effects data that had been used in the systematic review. Papers that had been included in more than one systematic review were only included once.

The first stage of the analysis was to check whether each paper was contained in MEDLINE and/or Embase, and this was done by using several search iterations as necessary using author names and/or words from the title.

### 2.3 Adverse effects terms in the database records

For each database, the available records were checked by 2 reviewers (S.G. and Y.L.) to ascertain if

1. The authors mentioned terms synonymous with “adverse effects” in the title or abstract, potentially enabling the paper to be found in an electronic search. Adverse effects terms, such as “adverse events,” “side effects,” “tolerated,” and “unwanted effects” were accepted.
2. The authors mentioned specific named adverse effects terms (such as “headache” or “cancer”) in the title or abstract. The terms were accepted based on the adverse effects included in the systematic review. For example, for a systematic review on cancer as an adverse effect, only cancer‐related terms were accepted. This part of the analysis was only conducted on included studies from reviews for a specific named adverse effect. As specific named adverse effects may be unknown to the searcher of a safety profile review at the time of searching.
3. The papers had been indexed (using subject headings or subheadings) with relevant terms for adverse effects, potentially enabling the paper to be found in an electronic search. Adverse effects terms were accepted on the basis that they could be considered synonymous with “adverse effects” such as side effects or “adverse effects (ae).”
4. The papers had been indexed with specific named adverse effects terms. The terms were accepted based on the adverse effects included in the systematic review. For example, for a systematic review on cancer as an adverse effect, only cancer‐related terms were accepted. This part of the analysis was only conducted on included studies from reviews for a specific named adverse effect.

Any disagreements between the reviewers as to whether a term was synonymous with “adverse effects” or a specific named adverse effect were resolved by discussion and consensus. We also referred to previously accepted terms used in research on systematic reviews of drug interventions.^5^, ^16^

### 2.4 Analysis

The percentage of papers identified via adverse effects terms in the title and abstract or indexing were recorded for MEDLINE alone, Embase alone, or a combination of both MEDLINE and Embase. In reviews of specific named adverse effects, the results were calculated separately with the use of specific named adverse effects and generic adverse effects terms as well as a combination of both. This enabled a comparison between MEDLINE and Embase, between textword and indexing search terms, and between generic and specific named adverse effect terms.

The results were calculated as a whole for all the included papers in the systematic reviews and again for each individual systematic review. This enabled any patterns of retrieval and any variance between different systematic reviews to be identified. The systematic reviews were categorised depending on the intervention evaluated.

To ascertain whether a search filter could be feasible, we used the measure of the percentage of relevant papers identified using a combination of all available adverse effects terms (also known as sensitivity). In general, the acceptable level of sensitivity of combinations of adverse effects search terms or a search filter is subjective and dependent on the topic of the search.^6^ However, for systematic review purposes, acceptable sensitivity tends to be defined as greater than 90%.^14^ We therefore used this threshold in our analysis.

The results were then compared with previous studies that evaluated the presence of adverse effects search terms for drug interventions.^5^, ^16^ To ascertain whether a search filter for nondrugs could be comparable in performance to drug adverse effects filters.

## 3 RESULTS

### 3.1 Systematic reviews included

**Figure 1:**
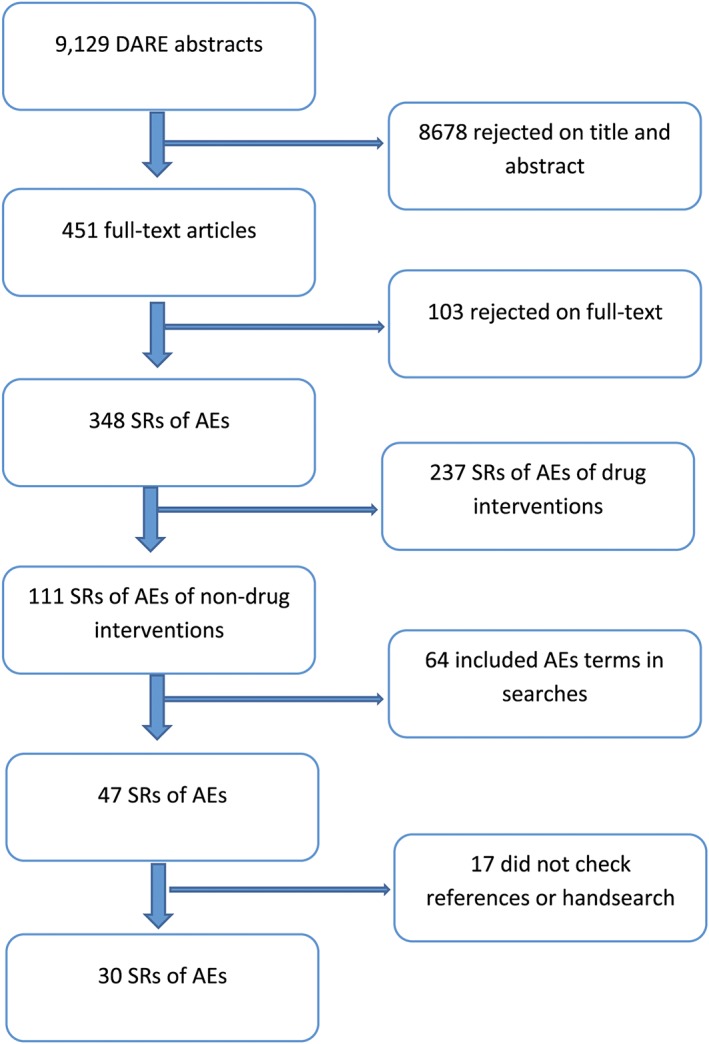
Flow diagram [Colour figure can be viewed at wileyonlinelibrary.com]

From 9129 DARE records screened, 451 full reports were retrieved and of these, 348 reviews were about adverse effects with 111 evaluating nondrug interventions. Sixty‐four of these 111 reviews were excluded as they did not include any adverse effects terms in their search strategies, and 17 of these were excluded as they did not check references lists or conduct hand searches. Thirty systematic reviews, therefore, met our inclusion criteria (Figure 1: flow diagram). Nineteen of the reviews evaluated a surgical intervention and 11 evaluated a nonsurgical intervention. As nearly two‐thirds of the reviews were of surgical interventions, the papers from the surgical and nonsurgical reviews were analysed separately. The nonsurgical interventions varied; 4 reviews were of physical therapy—(such as weight training or tai chi), 4 of physical interventions—(such as acupuncture or radiation therapy), 2 were of dentistry and 1 of a medical device (catheters).

The majority of the reviews (77%, 23/30) could be considered hypothesis generating reviews whereby the broad safety profile of an intervention was considered, rather than focusing down on a narrow set of particular outcomes. Six reviews (5 of which were of surgical interventions) considered a pre‐specified set of adverse effects (such as neurological, cardiovascular and cerebrovascular events, genitourinary or biliary complications, or infections). One review (of a surgical intervention) evaluated a specific pre‐specified adverse effect—perforation.

### 3.2 Primary studies included

A total of 644 papers were included in the 30 systematic reviews (range 3 to 86 papers per review). Nine included studies, however, were duplicate papers (contained in two systematic reviews), leaving 635 studies (358 from surgical intervention reviews and 277 from other nondrug reviews) for analysis.

### 3.3 Availability in MEDLINE and Embase

Of the 635 studies, 26 were not available on MEDLINE and 32 were not available in Embase. Of the 609 papers available in MEDLINE, 352 were from surgical intervention reviews and 257 from nonsurgical reviews. Of the 603 papers available on Embase, 348 were from surgical intervention reviews and 255 from nonsurgical reviews.

Each MEDLINE and Embase record was examined for the existence of adverse effects terms in the title, abstract, or indexing. A list of accepted terms identified in at least one paper is given in Table S1.

### 3.4 Average percentage of papers with adverse effects terms per systematic review

The average percentage of papers per systematic review containing adverse effects terms in the title, abstract, or indexing is summarised in Table 1 (details for individual systematic reviews are listed in Tables S2 and S3).

**Table 1:** Average percentage of records per systematic review with adverse effects terms

Intervention (Number of Systematic Reviews)	Type of Adverse Effects Terms (Number of Systematic Reviews)	Title or Abstract Adverse Effects Terms (Range)	Indexing Adverse Effect Terms (Range)	Title, Abstract, or Indexing Adverse Effects Terms (Range)
Surgical (n = 19)	Generic (n = 19)	MEDLINE		
		77% (27%‐100%)	63% (33%‐100%)	88% (47%‐100%)
		Embase		
		78% (33%‐100%)	73% (47%‐100%)	89% (47%‐100%)
	Specific (n = 6)	MEDLINE		
		60% (11%‐100%)	35% (0%‐82%)	64% (11%‐100%)
		Embase		
		61% (16%‐100%)	59% (16%‐100%)	66% (22%‐100%)
	Generic or specific (n = 19)	MEDLINE and Embase		
		82% (47%‐100%)	87% (53%‐100%)	94% (67%‐100%)
Nonsurgical (n = 11)	Generic (n = 11)	MEDLINE		
		48% (10%‐97%)	49% (5%‐97%)	66% (40%‐100%)
		Embase		
		47% (10%‐97%)	43% (15%‐82%)	66% (40%‐100%)
	Specific (n = 1)	MEDLINE		
		43%	17%	43%
		Embase		
		39%	45%	55%
	Generic or specific (n = 11)	MEDLINE and Embase		
		50% (10%‐97%)	60% (20%‐97%)	72% (40%‐100%)

While the percentage of adverse effects terms in the title and abstract in MEDLINE and Embase is similar, the percentage of records in Embase with indexing terms for adverse effects terms is generally higher (Table 1). In addition, it can be seen from Table 1 that papers for surgical interventions are more likely to contain terms for adverse effects than papers for nonsurgical interventions. Generic adverse effects terms also captured a higher percentage of papers than specific adverse effects terms.

Table 2 summarises these results for a combined search of both MEDLINE and Embase and presents a comparative analysis with studies of drug interventions.^5^, ^16^ In respect to papers on surgical interventions, a combined search of both MEDLINE and Embase retrieved an average of 94% (67% to 100%) of the included studies available. This is a similar percentage to the most recent drug intervention study, 92% (43% to 100%).^5^

**Table 2:** Average percentage of records per systematic review with adverse effects terms

Intervention (Number of Systematic Reviews)	Title or Abstract Adverse Effects Terms in MEDLINE or Embase (Range)	Indexing Adverse Effect Terms in MEDLINE or Embase (Range)	Title, Abstract, or Indexing Adverse Effects Terms in MEDLINE or Embase (Range)
Drug^16^ (n = 4)	59% (49%‐69%)	64% (54%‐76%)	77% (69%‐83%)
Drug^5^ (n = 26)	69% (21%‐100%)	90% (14%‐100%)	92% (43%‐100%)
Surgical (present study) (n = 19)	82% (47%‐100%)	87% (53%‐100%)	94% (67%‐100%)
Nonsurgical (present study) (n = 11)	50% (10%‐97%)	60% (20%‐97%)	72% (40%‐100%)

In the case of nonsurgical interventions, a combined search of both MEDLINE and Embase retrieved an average of 72% (40% to 100%) of the included studies available. This is lower than in the most recent study on drug interventions, 92% (43% to 100%),^5^ and lower than the surgical interventions, 94% (67% to 100%) (Table 2). However, this is comparable to the older drug intervention study in which adverse effects search terms retrieved 77% of relevant records (range 69%‐83%).

Overall, a combined search using terms in the title, abstract, or indexing run in both MEDLINE and Embase would have failed to retrieve 28% of papers across all the nonsurgical systematic reviews and 6% of papers across the surgical systematic reviews.

### 3.5 Overall percentage of adverse effects terms

In addition to an analysis of the papers per systematic review with adverse effects terms, we also conducted an overall analysis of the cohort of records (Table 3).

**Table 3:** Adverse effects terms in the title, abstract, or indexing of records of all included studies in MEDLINE and Embase

Intervention	Database (Number of Records)	Title or Abstract	Indexing	Subheadings	Indexing or Subheadings	Any Search Field
		Generic adverse effects terms				
Surgical	MEDLINE (n = 352)	275 (78%)	99 (28%)	168 (48%)	222 (81%)	314 (89%)
	Embase (n = 348)	275 (79%)	180 (49%)	212 (61%)	262 (75%)	315 (90%)
Nonsurgical	MEDLINE (n = 257)	127 (49%)	8 (3%)	116 (45%)	120 (47%)	166 (65%)
	Embase (n = 255)	127 (50%)	36 (14%)	89 (35%)	104 (41%)	164 (64%)
		Specific adverse effects				
Surgical	MEDLINE (n = 208)	85 (41%)	39 (19%)			89 (43%)
	Embase (n = 208)	88 (42%)	81 (39%)			94 (45%)
Nonsurgical	MEDLINE (n = 21)	9 (43%)	4 (19%)			10 (48%)
	Embase (n = 22)	9 (41%)	10 (45%)			12 (55%)
		Generic or specific adverse effects terms				
Surgical	MEDLINE (n = 352)	292 (83%)	108 (31%)	168 (48%)	231 (65%)	324 (92%)
	Embase (n = 348)	288 (83%)	191 (55%)	212 (61%)	276 (79%)	319 (92%)
Nonsurgical	MEDLINE (n = 257)	129 (50%)	9 (4%)	116 (45%)	121 (47%)	167 (65%)
	Embase (n = 255)	129 (51%)	36 (14%)	89 (35%)	104 (41%)	164 (64%)

Generic adverse effects terms (such as side effect or adverse event) may be used in either systematic reviews of a safety profile of an intervention or reviews of a specific named adverse effect. Overall surgical intervention papers are more likely to contain generic adverse effects terms in the title and abstract and indexing (Table 3) (89% versus 66%).

The use of specific named adverse effects search terms could only be assessed in systematic reviews of specific named adverse effects. Overall specific adverse effects terms in title and abstract were similar in surgical and nonsurgical intervention papers and fairly low (Table 3).

Using any adverse effects terms in the title, abstract, or indexing would have identified 92% of relevant references in MEDLINE and 92% in Embase for papers on surgical intervention but only 65% in MEDLINE and 64% in Embase of papers on nonsurgical interventions (Table 3).

## 4 DISCUSSION

We found substantial differences in the prevalence of adverse effects terms in studies of nondrug interventions. While the majority of adverse effects studies on surgical interventions can be retrieved by using both Embase and MEDLINE, over a quarter of adverse effect studies on nonsurgical interventions are likely to be missed with this method. Adverse effect terms performed better in studies of surgical interventions compared to those of nonsurgical interventions. Studies of surgical interventions were more likely to have free text terms such as *complication, complications*, *safe*, *safely*, and *safety*, or indexing terms such as *complication* (EMTREE), *patient safety* (EMTREE), and *safety* (EMTREE) and floating subheadings such as *adverse effects (ae)* and *complications (co)* in MEDLINE and *complication (co)* in Embase. The overall figures for the surgical intervention studies were close to that seen with recent studies of drug interventions. In contrast, nondrug, nonsurgical interventions had a much lower occurrence of generic adverse effects related terms. This has important implications for development of search filters of nondrug interventions or the use of adverse effects terms in nondrug reviews.

These results suggest that a search filter for surgical adverse effects may be feasible. As adverse effects terms are frequently present in the bibliographic records, search strategies could be successfully formulated using text mining solutions.

We were also able to investigate the use of generic and specific terms and using particular search fields when searching. Interestingly, the records of studies of surgical interventions and nonsurgical interventions both contained similar low levels of specific adverse effects terms in the title, abstract, or indexing (among studies from reviews of a specific adverse effect). This suggests that using specific adverse effects (without generic adverse effects) may only capture approximately half of all the relevant records. Reassuringly generic adverse effect terms performed much better. However, as searching for generic and specific search terms gave a higher yield than searching for either alone, a sensitive search should where possible contain both types of terms. In addition, sensitive searching for adverse effect should use terms in title/abstract and indexing as this gave a higher yield than searching in title/abstract or indexing alone.

It is debatable whether the creation of a generic adverse effects search filter covering all nondrug interventions is either feasible or useful. The sensitivity of such a filter is unlikely to reach acceptable levels for a systematic review. Filters categorised more specifically by type of intervention, however, may be feasible. For most types of interventions, the number of primary papers we identified was too small to assess the feasibility of a search filter. For example, there was only 30 included papers (from one systematic review) that evaluated medical devices. There was, however, 358 included papers on surgical interventions from 19 systematic reviews. The results suggest that a filter specifically targeted at adverse effects of surgical interventions may be plausible, given that a sensitivity of over 90% may be achievable.

The reasons for the lack of search terms for nonsurgical nondrug reviews may lie in the considerable diversity of nonsurgical, nondrug interventions such as dentistry, medical devices, physical interventions, and physical activity. This is very likely to lead to lack of standardisation of terms among the researchers. Also, adverse effects may not be considered to be an important part of those research areas, and so the terms are not given any prominence in the title, abstract, or indexing stages. Equally, the interventions may be perceived as very safe, and so only small amount of adverse effects are reported and subsequently indexed.

### 4.1 Limitations

The limited number of systematic reviews meeting our inclusion criteria means that it is not feasible to explore the feasibility of further search filters for different types of interventions. Another limitation is that the reliability of the results in this study is dependent to some extent on the quality of the search processes and sifting conducted within each included systematic review, which is difficult to judge. However, each review met the DARE quality inclusion criteria and had reported their search strategy in enough detail to ascertain that no adverse effects terms had been used and whether handsearching or reference checking had been conducted. The handsearching or reference checking may also help compensate for any limitations in the search strategies.

We excluded specific named adverse effects that are frequently related to surgery, for example, postoperative infection, postoperative pain, surgical infection, and surgical wound infection unless specified as the focus of the systematic review. If these terms were to be included, this may increase the sensitivity of the surgical intervention searches further.

## 5 CONCLUSION

Development of search filters for the adverse effects of surgical interventions with acceptable sensitivity for systematic reviews is a possibility. A future research priority should be to create search filters for adverse effects of surgical interventions in both MEDLINE and Embase.

The feasibility of developing filters for other types of nonsurgical interventions, such as medical devices, should also be undertaken using a much bigger sample size.

### 5.1 What is already known

Searching for adverse effects of nondrug interventions is difficult.

### 5.2 What is new

Searching for adverse effects of surgical interventions (with over 90% sensitivity) is feasible with adverse effects terms.

Searching for adverse effects of nonsurgical nondrug interventions (with over 90% sensitivity) is not feasible with adverse effects terms; however, more research is needed into specific interventions, such as medical devices.

### 5.3 Potential impact for RSM readers outside the authors' field

Searches for the adverse effects of surgical interventions could be made easier by the development of a search filter.

The addition of search terms for adverse effects of surgical interventions to searches could make the number of records to screen for a systematic review more manageable and still retrieve over 90% of the relevant papers.

## CONFLICTS OF INTEREST

S.G. had support from the National Institute for Health Research (NIHR) for the submitted work; S.G., K.W., and Y.L. had no financial relationships with any organisations that might have an interest in the submitted work; S.G., K.W., and Y.L. had no other relationships or activities that could appear to have influenced the submitted work.

## Supporting information

Table S1: List of accepted terms identified in at least one paper
Table S2: Records in each surgical review with ‘adverse effects’ terms in the title, abstract or indexing in MEDLINE or Embase
Table S3: Records in each non‐surgical review with ‘adverse effects’ terms in the title, abstract or indexing in MEDLINE or Embase
