# Supplementary material for: The feasibility of a search filter for the adverse effects of nondrug interventions in MEDLINE and Embase
Source: Res Synth Methods. 2017 Oct 11;8(4):506–13. doi: 10.1002/jrsm.1267 (PMC5725700; doi:10.1002/jrsm.1267)
Supplement: Supplementary file 1 — Table S1: List of accepted terms identified in at least one paper Table S2: Records in each surgical review with ‘adverse effects’ terms in the title, abstract or indexing in MEDLINE or Embase Table S3: Records in each non‐surgical review with ‘adverse effects’ terms in the title, abstract or indexing in MEDLINE or Embase [file JRSM-8-506-s001.docx]

# Supplementary Tables

## Table S1: List of accepted terms identified in at least one paper

| **MEDLINE** | **Embase** |
| --- | --- |
| **Surgical Interventions** |  |
| **Title or abstract (N=351)** | **Title or abstract (N=348)** |
| **GENERIC:** complications (208), safe (92), complication (77), safety (50), safely (16), adverse adj4 events (9), adverse events (8), procedure-related (8), postoperative morbidity (7), tolerated (7), risks (6), sequelae (3), safer (2), adverse effect (1), adverse event (1), adverse outcomes (1), adverse adj2 consequences (1), adverse adj2 outcomes (1) *(adverse cerebrovascular and cardiac events, adverse oncological consequences, adverse oncologic outcomes)* | **GENERIC:** complications (206), safe (90), complication (75), safety (50), safely (16), procedure-related (8), adverse events (8), adverse adj4 events (9), postoperative morbidity (7), risks (7), tolerated (7), sequelae (3), safer (2), adverse effect (1), adverse event (1) adverse outcomes (1), adverse adj2 consequences (1), adverse adj2 outcomes (1) *(adverse cerebrovascular and cardiac events, adverse oncological consequences, adverse oncologic outcomes)* |
| **SPECIFIC:**  bile duct injuries (2), biliary leak (2),biliary complication (1), biliary complications (1), bile duct injury (1), biliary leakage (1) *(N=70, Allemann 2014),* endoleak (4), renal infarction (4), serum creatinine (4), renal function (3), dialysis (2), renal infarcts (1), renal failure (1), hemodialysis (1), lost renal mass (1) *(N=5, Antoniou 2014),* wound infection (10), wound infections (6), site infection (3), site infections (2) *(N=15, Li 2014)*  mortality (14), death (6), stroke (9), bleeding (3), myocardial infarction (3), cerebrovascular events (2), cerebrovascular accidents (1), cerebrovascular and cardiac events (1), blood transfusion (1), *(N=17, Panchal 2014),* perforation (40) (N=85, van Halsema 2014), sensory loss (2), motor nerve loss (1), paresthesia (1) *(N=14, Varela Centelles 2014)* | **SPECIFIC:**  bile duct injuries (2), biliary leak (2), biliary complication (1), biliary complications (1), bile duct injury (1), biliary leakage (1) *(N=67, Allemann 2014),* endoleak (4),renal infarction (4), serum creatinine (4), dialysis (2), renal infarcts (1), renal failure (1), hemodialysis (1), renal function (3), lost renal mass (1) *(N=5, Antoniou 2014),* wound infection (10), wound infections (6), site infection (3), site infections (2),(N=15, Li 2014), mortality (14), stroke (9), death (6), bleeding (3), myocardial infarction (3), cerebrovascular events (2), cerebrovascular accidents (1), cerebrovascular and cardiac events (1), blood transfusion (1) *(N=17, Panchal 2014),* perforation (39) *(N=85, van Halsema 2014),* sensory loss (2), motor nerve loss (1) paresthesia (1) *(N=15, Varela Centelles 2014)* |
| **MeSH (N=351)** | **EMTREE (N=348)** |
| **GENERIC:** adverse effects (ae) (119), Postoperative Complications/ (96), complications (co) (81), Intraoperative Complications/ (5), patient safety/ (2), safety/ (2), equipment safety/ (1) | **GENERIC:** complication (co) (201), postoperative complication/ (120), complication/ (81), safety/ (42), patient safety/ (30), Complications/ (14), surgical risk/ (11), Adverse Drug Reaction (ae) (13), peroperative complication/ (7), perioperative complication/ (3), side effect (si) (2) |
| **SPECIFIC:** None identified *(N=70, Allemann 2014)*, Kidney Diseases/ (1) *(N=5, Antoniou 2014)*, Surgical Wound Infection/ (12) *(N=15, Li 2014)*, Mortality (mo) (12), stroke/ (6), survival rate/ (4), Hemorrhage/ (2), Ischemic Attack, Transient/ (1), Myocardial Infarction/ (1) *(N=17, Panchal 2014),* Intestinal Perforation/ (10) (N=85, van Halsema 2014), paresthesia/ (1), hypesthesia/ (1) *(N=14, Varela Centelles 2014)* | **SPECIFIC:** bile duct injury/ (4) *(N=67, Allemann 2014)*, endoleak/ (3), kidney function/ (3),kidney infarction/ (3), hemodialysis/ (1) *(N=5, Antoniou 2014),* wound infection/ (11), surgical infection/ (2), surgical site infection/ (1), *(N=15, Li 2014)*  mortality/ (14) , stroke/ (7), death/ (6), heart atrium fibrillation/ (5), surgical mortality/ (5), survival rate/ (4), bleeding/ (4), cerebrovascular accident/ (3), blood transfusion/ (2), transient ischemic attack/ (2), cardiovascular mortality/ (1), heart block/ (1), atrioventricular block/ (1), cerebrovascular disease/ (1), aorta rupture/ (1), coronary artery disease/ (1), blood vessel injury/ (1) *(N=17, Panchal 2014),* perforation/ (40) *(N=85, van Halsema 2014),* paresthesia/ (2), hypesthesia/ (1), sensory dysfunction/ (1) *(N=15, Varela Centelles 2014)* |
| **Non-surgical reviews** | |
| **Title or abstract (N=257)** | **Title or abstract (N=255)** |
| **GENERIC:** complications (55), safe (27), complication (21), adverse adj2 events (20), adverse events (19), safety (17), toxicity (14), tolerated (11), adverse effects (9), sequelae (7), adversely affect (4), adverse effect (4), risks (3), safely (3), side effects (3), safer (2), toxicities (2), adverse adj2 outcomes (2) adversely influence (1), affect adversely (1) adverse event (1), adverse impact (1), adverse outcomes (1), *(Adverse* cerebrovascular  *events (1), adverse pulp outcomes (1))* | **GENERIC:** complications (56), safe (27), complication (21), adverse events (18), safety (17), toxicity (14), tolerated (11), adverse effects (9), sequelae (6), adverse effect (4), adversely affect (3), risks (3), safely (3), side effects (3), safer (2), toxicities (2), adverse adj2 events (2), affect adversely (1), adverse event (1), adverse impact (1), adverse outcomes (1), adverse adj2 outcomes (2), *(Adverse* cerebrovascular *events (1), adverse pulp outcomes (1))* |
| **SPECIFIC:** pneumonia (3), infections (2), infectious (2), sepsis (2), infection (1), meningitis (1) *(N=21, Geurts 2014)* | **SPECIFIC:** pneumonia (3), infections (2), infectious (2), sepsis (2), infection (1), meningitis (1) *(N=22, Geurts 2014)* |
| **MeSH (N=257)** | **EMTREE (N=255)** |
| **GENERIC:** adverse effects (ae) (91), complications (co) (36), Postoperative Complications/ (6), safety/ (2) | **GENERIC:** complication (co) (76), adverse drug reaction (ae) (17), safety/ (15), complication/ (11), side effects (si) (8), adverse drug reaction/ (4), patient safety/ (4), side effect/ (4), complications/ (1), |
| **SPECIFIC:** Infection/ (2), Pneumonia/ (2) *(N=22, Geurts 2014)* | **SPECIFIC:** Pneumonia/ (5), infection/ (4), lung infection/ (3), sepsis/ (2), infection risk/ (1), infectious complication/ (1), meningitis/ (1) *(N=22, Geurts 2014)* |

### Table S2: Records in each surgical review with ‘adverse effects’ terms in the title, abstract or indexing in MEDLINE or Embase

| **Systematic review** | **Surgical Intervention** | **‘Adverse effects’ terms** | **Database(s)** | **Records available** | **AEs terms in title or abstract** | **AEs indexing** | **Retrievable by combined search** |
| --- | --- | --- | --- | --- | --- | --- | --- |
| Allemann 2014  (n=71) | Single-port laparoscopic cholecystectomy | Generic | MEDLINE | 70 | 61 (87%) | 31 (44%) | 65 (93%) |
|  |  |  | Embase | 67 | 59 (88%) | 54 (81%) | 64 (96%) |
|  |  | Specific: Biliary complications | MEDLINE | 70 | 8 (11%) | 0 | 8 (11%) |
|  |  |  | Embase | 67 | 8 (12%) | 4 (6%) | 9 (13%) |
|  |  | Generic and specific | MEDLINE | 70 | 61 (87%) | 31 (44%) | 65 (93%) |
|  |  |  | Embase | 67 | 59 (88%) | 54 (81%) | 64 (96%) |
|  |  |  | MEDLINE or Embase | 70 | 61 (87%) | 56 (80%) | 66 (94%) |
| Antoniou 2014 (n=5) | Endovascular aortic aneurysm repair | Generic | MEDLINE | 5 | 5 (100%) | 3 (60%) | 5 (100%) |
|  |  |  | Embase | 5 | 5 (100%) | 3 (60%) | 5 (100%) |
|  |  | Specific: Renal impairment | MEDLINE | 5 | 5 (100%) | 1 (20%) | 5 (100%) |
|  |  |  | Embase | 5 | 5 (100%) | 4 (80%) | 5 (100%) |
|  |  | Generic and specific | MEDLINE | 5 | 5 (100%) | 3 (60%) | 5 (100%) |
|  |  |  | Embase | 5 | 5 (100%) | 4 (80%) | 5 (100%) |
|  |  |  | MEDLINE or Embase | 5 | 5 (100%) | 5 (100%) | 5 (100%) |
| Bartel 2014 (n=8) | Achillon mini-open suture system | Generic | MEDLINE | 8 | 6 (75%) | 3 (38%) | 7 (88%) |
|  |  |  | Embase | 8 | 6 (75%) | 6 (75%) | 8 (100%) |
|  |  |  | MEDLINE or Embase | 8 | 6 (75%) | 7 (88%) | 8 (100%) |
| Jackson 2014 (n=9) | Robotic thyroidectomy | Generic | MEDLINE | 9 | 9 (100%) | 6 (67%) | 9 (100%) |
|  |  |  | Embase | 9 | 9 (100%) | 7 (78%) | 9 (100%) |
|  |  |  | MEDLINE or Embase | 9 | 9 (100%) | 9 (100%) | 9 (100%) |
| Lang 2014 (n=11) | Robotic-assisted thyroidectomy & conventional open thyroidectomy | Generic | MEDLINE | 11 | 7 (64%) | 7 (64%) | 9 (82%) |
|  |  |  | Embase | 11 | 7 (64%) | 7 (64%) | 10 (91%) |
|  |  |  | MEDLINE or Embase | 11 | 7 (64%) | 10 (91%) | 10 (91%) |
| Li 2014  (n=15) | Circular closure | Generic | MEDLINE | 15 | 10 (67%) | 10 (67%) | 12 (80%) |
|  |  |  | Embase | 15 | 10 (67%) | 7 (53%) | 12 (80%) |
|  |  | Specific: Surgical site infection | MEDLINE | 15 | 13 (87%) | 12 (80%) | 15 (100%) |
|  |  |  | Embase | 15 | 13 (87%) | 13 (87%) | 14 (93%) |
|  |  | Generic and specific | MEDLINE | 15 | 15 (100%) | 15 (100%) | 15 (100%) |
|  |  |  | Embase | 15 | 14 (93%) | 14 (93%) | 15 (100%) |
|  |  |  | MEDLINE or Embase | 15 | 15 (100%) | 15 (100%) | 15 (100%) |
| Liu 2014  (n=6) | Colonic stenting & emergency surgery | Generic | MEDLINE | 6 | 4 (67%) | 3 (50%) | 4 (67%) |
|  |  |  | Embase | 6 | 4 (67%) | 3 (50%) | 5 (83%) |
|  |  |  | MEDLINE or Embase | 6 | 4 (67%) | 4 (67%) | 5 (83%) |
| Panchal 2014 (n=17) | Transcatheter aortic valve implantation & surgical aortic valve replacement | Generic | MEDLINE | 17 | 12 (71%) | 12 (71%) | 15 (88%) |
|  |  |  | Embase | 17 | 12 (71%) | 14 (71%) | 15 (88%) |
|  |  | Specific: Mortality, major cardiovascular & cerebrovascular events | MEDLINE | 17 | 16 (94%) | 14 (82%) | 16 (94%) |
|  |  |  | Embase | 17 | 16 (94%) | 17 (100%) | 17 (100%) |
|  |  | Generic and specific | MEDLINE | 17 | 17 (100%) | 15 (88%) | 17 (100%) |
|  |  |  | Embase | 17 | 16 (94%) | 17 (100%) | 17 (100%) |
|  |  |  | MEDLINE or Embase | 17 | 17 (100%) | 17 (100%) | 17 (100%) |
| Simonson 2014  (n=15) | Ankle arthroscopy | Generic | MEDLINE | 15 | 5 (33%) | 10 (67%) | 13 (87%) |
|  |  |  | Embase | 15 | 5 (33%) | 7 (47%) | 10 (67%) |
|  |  |  | MEDLINE or Embase | 15 | 5 (33%) | 10 (67%) | 13 (87%) |
| Sodergren 2014  (n=14) | Transvaginal hybrid NOTES Cholecystectomy | Generic | MEDLINE | 14 | 12 (86%) | 5 (36%) | 12 (86%) |
|  |  |  | Embase | 14 | 12 (86%) | 11 (79%) | 12 (86%) |
|  |  |  | MEDLINE or Embase | 14 | 12 (86%) | 12 (86%) | 12 (86%) |
| Su 2014  (n=7) | Laparoscopic approach | Generic | MEDLINE | 7 | 7 (100%) | 5 (71%) | 7 (100%) |
|  |  |  | Embase | 7 | 7 (100%) | 6 (86%) | 7 (100%) |
|  |  |  | MEDLINE or Embase | 7 | 7 (100%) | 6 (86%) | 7 (100%) |
| Tsoi 2014 (n=14) | Breast reconstruction | Generic | MEDLINE | 14 | 11 (79%) | 8 (57%) | 12 (86%) |
|  |  |  | Embase | 14 | 11 (79%) | 10 (71%) | 12 (86%) |
|  |  |  | MEDLINE or Embase | 14 | 11 (79%) | 11 (79%) | 12 (86%) |
| van Halsema 2014  (n=86) | Colorectal stenting | Generic | MEDLINE | 86 | 73 (85%) | 71 (83%) | 82 (95%) |
|  |  |  | Embase | 85 | 74 (87%) | 70 (82%) | 81 (95%) |
|  |  | Specific: Perforation | MEDLINE | 86 | 40 (47%) | 10 (12%) | 41 (48%) |
|  |  |  | Embase | 85 | 43 (51%) | 40 (48%) | 45 (53%) |
|  |  | Generic and specific | MEDLINE | 86 | 78 (91%) | 72 (84%) | 85 (99%) |
|  |  |  | Embase | 85 | 77 (91%) | 73 (86%) | 82 (96%) |
|  |  |  | MEDLINE or Embase | 86 | 78 (91%) | 79 (92%) | 85 (99%) |
| Varela Centelles 2014  (n=16) | Lip biopsy | Generic | MEDLINE | 14 | 4 (27%) | 5 (33%) | 7 (47%) |
|  |  |  | Embase | 15 | 5 (33%) | 7 (47%) | 7 (47%) |
|  |  | Specific: Neurological events | MEDLINE | 14 | 3 (21%) | 2 (14%) | 4 (29%) |
|  |  |  | Embase | 15 | 3 (20%) | 3 (20%) | 4 (27%) |
|  |  | Generic and specific | MEDLINE | 14 | 6 (43%) | 5 (36%) | 8 (57%) |
|  |  |  | Embase | 15 | 7 (47%) | 7 (47%) | 7 (47%) |
|  |  |  | MEDLINE or Embase | 15 | 7 (47%) | 8 (53%) | 10 (67%) |
| Wang 2014  (n=24) | Gastrointestinal surgery | Generic | MEDLINE | 23 | 19 (83%) | 13 (56%) | 19 (83%) |
|  |  |  | Embase | 23 | 19 (83%) | 15 (65%) | 21 (91%) |
|  |  |  | MEDLINE or Embase | 23 | 19 (83%) | 16 (69%) | 21 (91%) |
| Wang 2014 (n=13) | Breast reconstruction | Generic | MEDLINE | 12 | 10 (91%) | 9 (82%) | 11 (%) |
|  |  |  | Embase | 11 | 9 (82%) | 10 (91%) | 11 (100%) |
|  |  |  | MEDLINE or Embase | 12 | 10 (91%) | 10 (91%) | 12 (100%) |
| Wormald 2014  (n=17) | Breast reconstruction | Generic | MEDLINE | 16 | 10 (63%) | 12 (75%) | 15 (94%) |
|  |  |  | Embase | 16 | 10 (63%) | 1 (88%) | 14 (88%) |
|  |  |  | MEDLINE or Embase | 16 | 10 (63%) | 16 (100%) | 16 (100%) |
| Xu 2014  (n=3) | Suprameatal approach & mastoidectomy with posterior tympanotomy approach in chochlear implantation | Generic | MEDLINE | 3 | 3 (100%) | 3 (100%) | 3 (100%) |
|  |  |  | Embase | 3 | 3 (100%) | 3 (100%) | 3 (100%) |
|  |  |  | MEDLINE or Embase | 3 | 3 (100%) | 3 (100%) | 3 (100%) |
| Zhang 2014 (n=16) | Liver transplantation | Generic | MEDLINE | 16 | 16 (100%) | 13 (81%) | 16 (100%) |
|  |  |  | Embase | 16 | 16 (100%) | 16 (100%) | 16 (100%) |
|  |  |  | MEDLINE or Embase | 16 | 16 (100%) | 16 (100%) | 16 (100%) |

### Table S3: Records in each non-surgical review with ‘adverse effects’ terms in the title, abstract or indexing in MEDLINE or Embase

| **SR (n=number of included studies)** | **Intervention** | **Type of ‘Adverse effects’ terms** | **Database(s)** | **Records available** | **AEs terms in title or abstract** | **AEs indexing** | **Retrievable by combined search** |
| --- | --- | --- | --- | --- | --- | --- | --- |
| Dentistry | | | | | | | |
| Dawson 2014  (n=10) | Composite resins | Generic | MEDLINE | 10 | 1 (10%) | 4 (40%) | 4 (40%) |
|  |  |  | Embase | 10 | 1 (10%) | 3 (30%) | 4 (40%) |
|  |  |  | MEDLINE or Embase | 10 | 1 (10%) | 4 (40%) | 4 (40%) |
| Quaranta 2014  (n=6) | Crown to implant ratio | Generic | MEDLINE | 6 | 3 (50%) | 3 (50%) | 5 (83%) |
|  |  |  | Embase | 6 | 3 (50%) | 3 (50%) | 5 (83%) |
|  |  |  | MEDLINE or Embase | 6 | 3 (50%) | 3 (50%) | 5 (83%) |
| Medical device | | | | | | | |
| Kulkarni 2014  (n=30) | Catheters | Generic | MEDLINE | 30 | 29 (97%) | 29 (97%) | 30 (100%) |
|  |  |  | Embase | 30 | 29 (97%) | 23 (77%) | 30 (100%) |
|  |  |  | MEDLINE or Embase | 30 | 29 (97%) | 29 (97%) | 30 (100%) |
| Physical intervention | | | | | | | |
| Geurts 2014  (n=23) | Therapeutic hypothermia | Generic | MEDLINE | 21 | 17 (81%) | 14 (61%) | 19 (90%) |
|  |  |  | Embase | 22 | 18 (82%) | 18 (82%) | 21 (95%) |
|  |  | Specific: Infection | MEDLINE | 21 | 9 (43%) | 4 (17%) | 10 (43%) |
|  |  |  | Embase | 22 | 9 (39%) | 10 (45%) | 12 (55%) |
|  |  | Generic and specific | MEDLINE | 21 | 19 (90%) | 15 (71%) | 20 (95%) |
|  |  |  | Embase | 22 | 20 (91%) | 18 (82%) | 21 (95%) |
|  |  |  | MEDLINE or Embase | 22 | 21 (95%) | 19 (90%) | 21 (95%) |
| Ishiyama 2014  (n=32) | Radiotherapy | Generic | MEDLINE | 32 | 24 (75%) | 24 (75%) | 31 (97%) |
|  |  |  | Embase | 32 | 24 (75%) | 22 (69%) | 27 (84%) |
|  |  |  | MEDLINE or Embase | 32 | 25 (78%) | 27 (84%) | 31 (97%) |
| Park 2014  (n=26) | Acupuncture | Generic | MEDLINE | 20 | 8 (40%) | 1 (5%) | 8 (40%) |
|  |  |  | Embase | 20 | 8 (40%) | 6 (30%) | 10 (50%) |
|  |  |  | MEDLINE or Embase | 20 | 8 (40%) | 7 (35%) | 10 (50%) |
| Wang 2014  (n=23) | Blood transfusion | Generic | MEDLINE | 18 | 8 (44%) | 13 (72%) | 15 (83%) |
|  |  |  | Embase | 17 | 7 (41%) | 6 (35%) | 11 (65%) |
|  |  |  | MEDLINE or Embase | 18 | 8 (44%) | 15 (83%) | 16 (89%) |
| Physical Activity | | | | | | | |
| Paramanandam 2014 (n=11) | Weight training | Generic | MEDLINE | 11 | 4 (36%) | 7 (64%) | 7 (64%) |
|  |  |  | Embase | 11 | 4 (36%) | 5 (45%) | 8 (73%) |
|  |  |  | MEDLINE or Embase | 11 | 4 (36%) | 9 (82%) | 9 (82%) |
| Pilutti 2014  (n=26) | Exercise training | Generic | MEDLINE | 24 | 7 (29%) | 8 (31%) | 10 (42%) |
|  |  |  | Embase | 23 | 6 (26%) | 5 (22%) | 10 (43%) |
|  |  |  | MEDLINE or Embase | 25 | 7 (28%) | 9 (36%) | 13 (52%) |
| Warms 2014  (n=38) | Cardiovascular-related training programs | Generic | MEDLINE | 36 | 13 (36%) | 11 (31%) | 16 (42%) |
|  |  |  | Embase | 37 | 13 (35%) | 6 (16%) | 16 (43%) |
|  |  |  | MEDLINE or Embase | 37 | 13 (35%) | 15 (41%) | 20 (54%) |
| Wayne  (n=52) | Tai chi | Generic | MEDLINE | 49 | 19 (39%) | 6 (12%) | 21 (43%) |
|  |  |  | Embase | 47 | 19 (40%) | 7 (15%) | 22 (47%) |
|  |  |  | MEDLINE or Embase | 49 | 19 (39%) | 10 (20%) | 22 (45%) |
